# Monitoring HPV16/18 immunisation in England and elsewhere

**DOI:** 10.1038/sj.bjc.6605758

**Published:** 2010-07-13

**Authors:** S Franceschi

**Affiliations:** 1 International Agency for Research on Cancer, 150 cours Albert Thomas, 69372 Lyon cedex 08, France

To be really useful, a vaccine must be highly efficacious, safe, and must be accepted by and administered to the majority of the target population. Current prophylactic vaccines against human papillomavirus (HPV), based on L1 virus-like particles, have been extensively evaluated for both efficacy against severe precancerous lesions of the cervix uteri and safety (Schiller *et al*, 2008). On account of a spectacular 100% efficacy in young women not infected with the HPV vaccine types (HPV16/18), the vaccines have been incorporated into national immunisation programmes in the United States and several European countries (Austria, France, Germany, Italy, and the United Kingdom) faster than any vaccine ever before. Immunisation against HPV in combination with cervical screening has the potential to eradicate cervical cancer.

Current HPV vaccines were the product of 100 years of epidemiological data on the association between cervical cancer and a sexually transmitted agent, and 20 years of firm biological evidence that a few mucosal HPV types (denominated oncogenic types) were such agents (IARC, 1995). Although some 20 different HPV types were known to infect the genital tract (IARC, 1995), vaccine research had focused since the beginning on two oncogenic HPV types (HPV16/18). Two non-oncogenic types (HPV6 and 11) were also intensively studied for the purpose of preventing genital warts. However, the decision to develop a vaccine against HPV16 (Koutsky *et al*, 2002), and subsequently HPV18, was made based on relatively few studies that were small in size, and that often tested for very few oncogenic HPV types other than HPV16/18 (IARC, 1995).

Information from over 30 000 invasive cervical cancers from five continents has now accumulated, and has not only confirmed the predominance of HPV16 (57%) and HPV18 (16%) in every studied population, but has also showed the similarity in the relative importance of other oncogenic types (HPV31, 33, 35, 45, 52, and 58) (Li *et al*, 2010).

In the present issue of the *British Journal of Cancer*, Howell-Jones *et al* provide information on the prevalence of oncogenic HPV types across the full spectrum of cervical pathology, from normal to cancer, in a nationally representative sample of 6234 women in England. The study, one of the largest and most accurate ever carried out, represents a robust baseline picture against which changes in HPV prevalence and type distribution following the introduction of the HPV Vaccination Programme in 2008 in England can be evaluated (Department of Health, 2010). The prevalence of HPV16 was low among women without cytological abnormalities (3%), but it greatly increased in women with cervical intraepithelial neoplasia grade 3 (CIN3) (57%), squamous cell carcinoma (66%), and adenocarcinoma (48%) of the cervix. Infection with HPV18 was rarer than HPV16 in women without cytological abnormalities (1%), and in those with CIN3 (8%), and squamous cell carcinoma (13%), but nearly equally frequent (40%) in women with adenocarcinoma.

Howell-Jones *et al* also emphasised the difficulties of attributing a cervical cancer or precancerous lesion to an individual HPV type in the presence of multiple-type infections. Concurrent detection of two HPV types or more has become increasingly frequent in the last 20 years (Li *et al*, 2010) on account of the use of more and more sensitive HPV tests (e.g. the Linear Array typing system (Roche Molecular Systems, Inc., Branchburg, NJ, USA) used by Howell-Jones *et al*). Immunisation against HPV16/18 in England has, therefore, the potential to prevent 56–65% of CIN3 and 73–77% of invasive cervical cancer. Finally, cross-protection against oncogenic types other than HPV16/18 may prevent an additional 3–4% of invasive cervical cancer.

The multi-site HPV prevalence study by Howell-Jones *et al* also highlighted the high frequency of infection with oncogenic HPV types among women in England. Prevalence was 16% among all screened women, but rose to 29% among women aged 25–29 years, which is as high as that found in countries like Colombia (Molano *et al*, 2002) and Chennai, India (Franceschi *et al*, 2005), where cervical cancer rates are 3–4-fold higher than in the United Kingdom (Ferlay *et al*, 2010). High-quality cervical screening is the clear explanation for the discrepancy between high HPV prevalence and low cervical cancer incidence in England (Peto *et al*, 2004).

Will HPV16/18 immunisation reduce cervical cancer incidence any further in England and elsewhere? A few unique features of HPV vaccines represent special challenges: (1) they are expensive vaccines; (2) they target adolescent girls for whom no delivery platform is readily available in any country; and (3) they are meant to prevent a cancer for which an effective secondary prevention strategy exists (Franceschi *et al*, 2009). High-quality screening can diminish cervical cancer much more quickly than immunisation against HPV, but it is complicated, very expensive and very inequitably distributed within and between world countries. The success of vaccine delivery in England gives good reason to be optimistic. In the first year of the HPV immunisation programme, 80% of 12–13-year-old girls (routine cohort) received all three HPV vaccine doses (Department of Health, 2010). Also importantly, national data show little evidence of inequality in coverage among 12-year-old girls by deprivation level of the areas where they live. This contrasts with a correlation with deprivation for cervical screening uptake even in England (Desai *et al*, 2010).

High-vaccine uptake was also reported in similar school-based programmes in Scotland (National Health Services Scotland, 2010), Wales (National Public Health Service for Wales, 2009), and Australia (Brotherton *et al*, 2008), but is in sharp contrast with low uptake in other high-resource countries where HPV16/18 vaccination is distributed by the private sector, regardless, to some extent, of reimbursement policies. In the United States, where vaccine delivery is mainly through family doctors, the uptake of more than one vaccine dose among 13–17-year-old girls was only 25% (Bach, 2010). In addition, at the state level, vaccination rates in the United States were strongly and inversely correlated with cervical cancer mortality rates and median income (Bach, 2010). Likewise, in the European Union, no HPV16/18 vaccination programme has been started in the poorest Member States, where no good cervical screening in place and where cervical cancer incidence rates are the highest (Ferlay *et al*, 2010). The introduction of HPV16/18 vaccination programmes in low- and medium-resource countries outside Europe is stalling, and is severely threatened by the economic downturn (Butler, 2010). If HPV16/18 immunisation fails to reach women and populations who are underserved by cervical screening, hardly any lives will be saved.
